# Associations between maternal depressive symptoms and child feeding practices in a cross-sectional study of low-income mothers and their young children

**DOI:** 10.1186/1479-5868-11-75

**Published:** 2014-06-16

**Authors:** Alison N Goulding, Katherine L Rosenblum, Alison L Miller, Karen E Peterson, Yu-Pu Chen, Niko Kaciroti, Julie C Lumeng

**Affiliations:** 1 University of Michigan Medical School, Ann Arbor, MI, USA; 2 Center for Human Growth and Development, University of Michigan, 300 North Ingalls Street, 10th Floor, 48109 Ann Arbor, MI, USA; 3 Department of Psychiatry, University of Michigan, Ann Arbor, MI, USA; 4 Department of Health Behavior and Health Education, University of Michigan School of Public Health, Ann Arbor, MI, USA; 5 Department of Environmental Health Sciences, University of Michigan School of Public Health, Ann Arbor, MI, USA; 6 Department of Nutrition, Harvard School of Public Health, Boston, MA, USA; 7 Department of Biostatistics, University of Michigan School of Public Health, Ann Arbor, MI, USA; 8 Department of Pediatrics, University of Michigan Medical School, Ann Arbor, MI, USA

**Keywords:** Depression, Mothers, Child, Mother-child relations, Feeding behavior

## Abstract

**Background:**

Maternal depression may influence feeding practices important in determining child eating behaviors and weight. However, the association between maternal depressive symptoms and feeding practices has been inconsistent, and most prior studies used self-report questionnaires alone to characterize feeding. The purpose of this study was to identify feeding practices associated with maternal depressive symptoms using multiple methodologies, and to test the hypothesis that maternal depressive symptoms are associated with less responsive feeding practices.

**Methods:**

In this cross-sectional, observational study, participants (*n* = 295) included low-income mothers and their 4- to 8-year-old children. Maternal feeding practices were assessed via interviewer-administered questionnaires, semi-structured narrative interviews, and videotaped observations in home and laboratory settings. Maternal depressive symptoms were measured using the Center for Epidemiologic Studies-Depression scale (CES-D). Regression analyses examined associations between elevated depressive symptoms (CES-D score ≥16) and measures of maternal feeding practices, adjusting for: child sex, food fussiness, number of older siblings; and maternal age, body mass index (BMI), education, race/ethnicity, single parent status, perceived child weight, and concern about child weight.

**Results:**

Thirty-one percent of mothers reported depressive symptoms above the screening cutoff. Mothers with elevated depressive symptoms reported more pressuring of children to eat (β = 0.29; 95% Confidence Interval (CI): 0.03, 0.54) and more overall demandingness (β = 0.16; 95% CI: 0.03, 0.29), and expressed lower authority in child feeding during semi-structured narrative interview (Odds Ratio (OR) for low authority: 2.82; 95% CI: 1.55, 5.12). In homes of mothers with elevated depressive symptoms, the television was more likely audible during meals (OR: 1.91; 95% CI: 1.05, 3.48) and mothers were less likely to eat with children (OR: 0.48; 95% CI: 0.27, 0.85). There were no associations between maternal depressive symptoms and encouragement or discouragement of food in laboratory eating interactions.

**Conclusions:**

Mothers with elevated depressive symptoms demonstrated less responsive feeding practices than mothers with lower levels of depressive symptoms. These results suggest that screening for maternal depressive symptoms may be useful when counseling on healthy child feeding practices. Given inconsistencies across methodologies, future research should include multiple methods of characterizing feeding practices and direct comparisons of different methodologies.

## Background

Recent studies have explored the potential association between maternal mental health and child obesity, with some suggesting a role for maternal depression in the development and maintenance of child obesity [1-4]. Depressed mothers demonstrate less engagement and more irritability towards their children in general [5]; if such problematic interaction styles extend to the realm of feeding, this represents a potential pathway through which maternal depression may influence child eating and weight status. Parents shape children’s eating behaviors in many ways [6], and suboptimal feeding practices may promote dysfunctional eating behaviors in children and contribute to child obesity [7,8]. Given that both depressive symptoms and feeding practices are modifiable, understanding their relationships to child eating and weight status may be important for clinicians and researchers who seek to reduce child obesity.

Some studies have linked maternal depressive symptoms to potentially detrimental feeding practices, including being less likely to set limits or restrict child intake [3], or being more likely to pressure children to eat [9,10] or to adopt forceful, indulgent, or uninvolved feeding styles [11]. A recent systematic review reported that such forms of non-responsive feeding (broadly defined as a lack of reciprocity in the feeding interaction where either the caregiver or child exerts excessive control) are associated with child overweight/obesity [12]. Yet not all studies find that mothers with depressive symptoms feed in a less responsive manner; others have found no significant associations between maternal depressive symptoms and any measures of pressuring or restrictive feeding practices [13-17].

Recent reviews on parental feeding practices highlight methodological limitations in the literature, including reliance of most prior studies on self-report questionnaires about feeding practices [12,18]. The questionnaire methodology has important limitations, including self-report bias and inconsistent question interpretation [19]. Furthermore, mother-reported feeding practices may not be associated with observed maternal feeding practices [20,21]. We were able to identify only two studies using non-questionnaire approaches to examine the association between maternal depressive symptoms and maternal feeding practices. Both studies used video observations to assess mothers’ feeding behaviors with their young children in laboratory-based feeding situations. While one study found an association between maternal depressive symptoms and controlling feeding practices [10], the other did not [17]. In addition, most studies on maternal mental health and feeding practices have examined homogenous samples [9,10,13-15,22,23]. More multi-method studies of diverse populations are needed.

The current study examines associations between maternal depressive symptoms and child feeding practices as characterized by interviewer-administered questionnaires, semi-structured narrative interviews, and video observation methods in home and laboratory settings in a population of low-income mothers of 4- to 8-year-old children. We sought to test the hypothesis that mothers with elevated depressive symptoms exhibit less responsive feeding practices than mothers with lower levels of depressive symptoms.

## Methods

### Subjects

The study population included 295 caregiver-child pairs recruited from Head Start programs (free, federally-subsidized preschool programs for low-income children) in Southeastern Michigan. Most participants were drawn from a longitudinal cohort established in 2009–2011, with some (n = 17) additional caregiver-child pairs recruited in May 2013 by flyers distributed to Head Start locations. For the original cohort, all families with children enrolled in regional Head Start programs were invited to participate in a study investigating associations between stress and eating in children. After completing the original study, primary caregivers were later contacted by phone and invited to participate in this follow-up study, which was described as a research study on children’s eating behaviors with parents. Those meeting the following criteria were eligible for inclusion: caregiver has less than a four-year college degree; caregiver fluent in English; child born at 35 weeks gestation or more and without significant perinatal or neonatal complications; child without history of food allergies, serious medical problems, or any form of disordered eating; child not in foster care. Because all child participants were originally recruited from Head Start programs, they were at the time of recruitment into the original study aged 3 to 4 years and living in poverty. This study included children from 4 to 8 years of age, focusing on middle childhood when children develop new capacities for acquiring and using information [24], and thus may be more strongly influenced by maternal feeding practices.

Of 301 primary caregivers enrolled in the current study, we excluded five male caregivers from analysis, and one female caregiver only completed the semi-structured narrative interview, resulting in a sample of 295 female primary caregivers and their children. Within this sample 95% were biological mothers, with the other 5% composed of adoptive mothers, stepmothers, and grandmothers; henceforth we refer to the entire group as “mothers”. All mothers gave written informed consent, and were each compensated $150 for participating in all study procedures. The University of Michigan Institutional Review Board approved this study.

### Overall study procedure

During the first study visit, the mother completed a depression scale, questionnaires about feeding practices, and the semi-structured narrative interview. Then the mother returned with her child for a second study visit, during which the standardized laboratory eating protocol was administered and the mother was weighed and measured. The mother also completed a questionnaire about her child’s eating behaviors during the second study visit. Given the prevalence of low literacy in our study population, research assistants administered all questionnaires to mothers using laptop computers, with the research assistant reading each question and its response options aloud and entering the mother’s responses. All questionnaires were administered in their entirety, but for our analysis we only examined selected scales (presented in Table 1). The videotaped observations took place at home, and each mother was asked to record three routine meals within one week.

**Table 1:** Description of feeding practices assessed using questionnaire scales

**Scale**	**Description**	**Sample item**
Perceived responsibility [28]	Mother’s perception of her level of responsibility for child feeding	“When your child is at home, how often are you responsible for feeding him/her?”^1^
Pressure to eat [28]	Mother’s tendency to pressure child to eat more food at meals	“My child should always eat all of the food on his/her plate”.^2^
Restriction [28]	Extent to which mother restricts child’s access to foods	“I have to be sure that my child does not eat too much of his/her favorite food”.^2^
Monitoring [28]	Extent to which mother oversees child eating	“How much do you keep track of the high-fat foods that your child eats?”^1^
Demandingness [29]	How much mother encourages or discourages child’s eating behaviors	“How often during the dinner meal do you tell the child to eat at least a little bit of food on his/her plate?”^1^
Perceived child weight [28]	Mother’s perceptions of child’s weight status history	“When your child was a toddler, was your child: markedly underweight, underweight, normal, overweight, or markedly overweight?”
Concern about child weight [28]	Extent of maternal concern for child becoming overweight	“How concerned are you about your child becoming overweight?”^3^
Food fussiness [42]	How selective child is with regard to trying new foods and enjoying a varied diet	“My child refuses new foods at first”.^1^

^1^Responses based on 1–5 Likert scale ranging from “never” to “always”.

^2^Responses based on 1–5 Likert scale ranging from “disagree” to “agree”.

^3^Responses based on 1–5 Likert scale ranging from “unconcerned” to “very concerned”.

### Primary predictor: maternal depressive symptoms

All mothers completed the Center for Epidemiologic Studies-Depression scale (CES-D), a valid, reliable 20-item questionnaire designed to measure depressive symptoms in the general population across a wide range of demographic characteristics [25]. CES-D scores range from 0 to 60, with higher scores indicating more severe depressive symptoms. The widely-employed threshold of CES-D score ≥ 16 was used to represent clinically significant depressive symptoms; this score represented the 80^th^ percentile in a reference population [26] and has been shown to agree well with longer self-report depression scales and clinician interview ratings [27].

### Primary outcome: maternal feeding practices

Maternal feeding practices were assessed via three methods: interviewer-administered questionnaires completed by the mother, semi-structured narrative interviews with the mother, and videotaped observations of maternal-child feeding situations in both home and laboratory settings.

#### Questionnaires

Interviewer-administered questionnaires to assess maternal feeding practices included the Child Feeding Questionnaire (CFQ) [28] and the Caregiver’s Feeding Styles Questionnaire (CFSQ) [29]. The CFQ is a 31-item measure with established reliability and validity [28,30]. We investigated the Perceived Responsibility (3 items, Cronbach’s α = 0.73), Pressure to Eat (4 items, Cronbach’s α = 0.62), Restriction (8 items, Cronbach’s α = 0.75), and Monitoring (3 items, Cronbach’s α = 0.86) scales from the CFQ. The CFSQ is a 19-item valid, reliable measure developed for use in the Head Start population [29]. We analyzed the Demandingness score, which represents the degree to which mothers encourage or discourage children’s eating and is calculated as the mean of all 19 items (Cronbach’s α = 0.85). All responses were scored on a 1–5 Likert scale, and mean scores were generated to produce summary scores for each scale. All 295 mothers completed the Perceived Responsibility, Pressure to Eat, and Monitoring questions from the CFQ; one mother did not answer multiple questions from the Restriction scale so this score could only be calculated for 294 mothers. All 295 mothers completed the CFSQ.

#### Semi-structured narrative interview

Each mother participated in a semi-structured narrative interview (mean length 46 minutes, range 21 to 148 minutes). Interviews were designed to elicit maternal narratives about feeding, and children were not present during the interviews. The interviews were administered by trained research assistants and consisted of open-ended questions (sample questions are presented in Table 2). Research assistants were trained to avoid giving positive, negative, or leading reactions to a mother’s answers. Interviews were audiotaped and transcribed by laboratory staff. A refined coding scheme slightly adapted from prior work [31] was applied to interview transcripts, allowing for reliable classification into each of three domains regarding maternal narratives about feeding: authority, confidence, and investment. The coding scheme is presented in Table 3. Twenty percent of the interviews were coded by two raters and inter-rater reliability was acceptable for all codes (Cohen’s κ 0.72, 0.89, and 0.69, respectively). All 295 participating mothers completed the interview, but two interviews were considered uncodeable due to mild limitations in fluent spoken English that were felt to invalidate the coding scheme.

**Table 2:** Examples of open-ended questions used to prompt maternal narratives about feeding their children

**Question**	**Follow-up question**
How do people in your house usually eat their meals on a typical day?	About mealtime, what works well and what does not?
Can you describe yesterday’s dinner?	And thinking about the dinner that you just described, how did you feel about it?
How do you know if [child name] is growing appropriately?	When do you seek advice on how to feed [child name]?
Do you ever worry that [child name] doesn’t or might not eat enough?	
Do you ever worry that [child name] does or might eat too much?	
Do you ever worry about the kinds of foods [child name] eats?	
How were you fed when you were growing up?	Thinking about how you were fed growing up, do you see similarities or differences to your own way of feeding your kids?
In your opinion, what causes a child to be overweight?	
Can you help me brainstorm some things parents can do to keep their children from becoming overweight?	Do you do any of these things?
What advice do you have for other parents about how to best feed their kids?	

**Table 3:** Coding scheme used to categorize maternal feeding styles with regard to authority, confidence, and investment

**Authority**	
High authority	Mother sets limits and structure for food and mealtime. She guides her child’s food choices and eating decisions pertaining to what, when, where, or how much is consumed. The mother is clearly in control and purposefully sets the agenda for feeding her child.
Low authority	Mother sets few or no expectations or rules surrounding feeding her child. She allows the child to drive decisions about what, when, where, or how much is consumed. Her manner may range from indulgent/loving to harsh/neglectful, but the key point is that the child sets the agenda for feeding.
**Confidence**	
Confident	Mother does not question her decisions about how she chooses to feed her child, and is confident that her child is eating properly. She may have a few doubts, but they do not overwhelm her strong feelings that she is doing the right thing and correctly addressing any child feeding challenges.
Not Confident	Mother has doubts and is unsure about how she feed her child. She may be confident in some aspects of feeding, but overall questions whether her child is being fed properly. She may express worry, uncertainty, or concern about how she is handling difficult child feeding situations.
**Investment**	
Deeply	There is a sense that love and concern is expressed through the mother’s feeding practices. She speaks passionately and has invested significant cognitive energy in feeding as demonstrated by expression of complex thoughts about feeding her child.
Mildly	Mother has given some thought to feeding her child, but she does not speak passionately and her interview is not characterized by significant affect. She may have a few complex thoughts about feeding, but lacks fervor and enthusiasm when describing her feeding practices.
Removed	Mother is relatively indifferent to child’s eating habits. She is unconcerned and shares limited thoughts related to feeding. She may present as neglectful, or may simply appear unconcerned about child feeding.

#### Videotaped observations of mother-child feeding situations

For the home meal observations, each mother was loaned a camera and asked to videotape three typical dinnertime meals over the course of one week. The mother was instructed to set up the camera so that the child’s upper torso, plate, and drink were always in view, and to record the entirety of each meal. For these home meal observations, we developed a novel coding scheme, based on prior approaches [32,33], to code (yes vs. no for each meal): child eating at kitchen/dining table, television audible, and mother eating with child for any portion of the meal. Coders were trained to reliability; 12% of videos were coded by two raters and inter-rater reliability by Cohen’s κ exceeded 0.70 for all codes. For analysis, we combined data from all available home meal observations associated with a single mother-child pair to create collapsed variables. We defined each outcome as occurring “always” vs. “not always,” where “always” referred to all available videotaped home meal observations for a mother-child pair. Of the 295 participating mother-child pairs, 234 (79%) submitted three codeable videos, 266 (90%) submitted two or more, and 270 (92%) submitted at least one.

In the laboratory, mother-child pairs were videotaped during a standardized food presentation protocol. This standardized approach is useful in examining mother-child response to novel foods (which are often not offered during typical family mealtimes at home), and also serves to reduce the broad variability that occurs at home (when mothers may be occupied attending to other children in the family, or children may be attending more to siblings or the television than engaging with their mothers around food). Mothers and children were asked to fast for two hours beforehand. To begin the protocol, they were seated alone at a table in a quiet room, and four foods were presented individually and sequentially in random order. For each food, the mother and child were given individual servings, invited to try the food using a standardized script, and then left alone for four minutes while videotaped, after which the food was removed and the next food presented. After each food, a research assistant briefly interviewed the mother and child about their opinions of the food and recorded responses on paper. The four foods differed in sweetness (dessert or vegetable) and familiarity (familiar or unfamiliar). We chose to focus on sweetness and familiarity because these characteristics are important predictors of food preferences in young children [34], and such preferences are critical in making long-term improvements in children’s diets [35]. The specific food items served to each individual included one cup of green beans (Del Monte, Cut Green Beans, No Salt Added, 123.7 ± 0.5 grams), one cup of artichoke hearts (Reese Quartered, Artichoke Hearts, 123.7 ± 0.5 grams), two cupcakes (Hostess Chocolate Cupcakes, 104.96 ± 0.5 grams), and one-quarter container of halva (Ziyad, Halva with Vanilla, 76 ± 0.5 grams). The portion sizes were identical for both mother and child, and were specifically selected to be large in order to elicit restrictive maternal feeding behaviors. All foods were prepared in advance and presented in plastic containers without brand packaging. Food was weighed on a scale (Scout Pro Balance Model SP202) with accuracy ± 0.01 gram before and after presentation.

A coding scheme based on the BATMAN (Bob and Tom’s Method of Assessing Nutrition) coding scheme [36] and used in prior work by the investigators was applied to video recordings of the laboratory protocol. The scheme counts number of verbal encouragements, physical encouragements, verbal discouragements, and physical discouragements. For the 20% of video recordings coded by two raters, inter-rater reliability was high, with intraclass correlation coefficients ranging from 0.81 to 1.00 for all codes. Physical encouragements or discouragements occurred very infrequently. Therefore for analysis, we collapsed physical and verbal encouragement to create a summary variable for total maternal encouragement, and similarly collapsed physical and verbal discouragement to create a summary variable for total maternal discouragement. Given that prior studies have only presented familiar foods during laboratory eating interactions [10,17], we collapsed data from green beans and cupcakes to create summary variables for familiar foods, and similarly collapsed artichoke hearts and halva data to create summary variables for unfamiliar foods. Since this task involved the potential consumption of novel foods, mother-child pairs were excluded if the mother had a history of an adverse reaction to a food, or if the child had experienced an adverse food reaction since initial enrollment. Of the 295 mother-child pairs enrolled in our study, 228 (77%) completed the laboratory food presentation protocol, with the majority (44/67 = 66%) excluded due to a history of adverse reactions to food.

### Covariates

Mothers reported sociodemographic information including: child sex, child birthdate, number of older siblings; and maternal birthdate, education, race/ethnicity, and single parent status. Child and maternal birthdates were used to calculate age by subtracting birthdate from date of the first study visit. One mother was unable to provide her birthdate. Maternal education was included as “≤ high school diploma or equivalent” vs. “> high school diploma”, with the highest educational level in this sample being less than a four-year college degree. Maternal race/ethnicity was included as “non-Hispanic white” vs. “Hispanic and/or non-white.” Single parent status was considered to be anything other than “married” or “in a committed relationship with a partner”.

Maternal weight and height were measured without shoes or heavy clothing according to a standardized procedure. Body Mass Index (BMI) for the mothers was calculated as weight in kilograms divided by height in meters squared. For mothers who were pregnant or had recently given birth (n = 44), self-reported pre-pregnancy weight was used instead of measured weight. BMI could not be calculated for two mothers who had given birth within three months of the study visit and did not know their pre-pregnancy weight.

Maternal concern about child weight has been associated with controlling feeding practices [37], and difficult child feeding behaviors have been associated with both controlling feeding practices [37] and maternal psychopathology [38,39]. We elected to focus on maternal perception of child weight status rather than child BMI because there is evidence that low-income mothers distrust growth charts and do not endorse BMI-based definitions of child overweight [40,41]. These factors were assessed using three questionnaire scales: 1) the Perceived Child Weight and 2) Concern about Child Weight scales from the CFQ; and 3) the Food Fussiness scale from the Child Eating Behavior Questionnaire (CEBQ), a validated and reliable 35-item questionnaire to assess eating style in children based on parental report [42]. The Perceived Child Weight scale (3 items, Cronbach’s α = 0.72) asks mothers to rate their children on a 1–5 Likert scale from “markedly underweight” to “markedly overweight” at different child ages. We created a 3-category variable for this analysis: underweight (“markedly underweight” or “underweight”), normal, or overweight (“markedly overweight” or “overweight”). The Concern about Child Weight scale (3 items, Cronbach’s α = 0.70) asks mothers to rate their concern on a 1–5 Likert scale from “unconcerned” to “very concerned” in regards to their children overeating or becoming overweight. The Food Fussiness scale (6 items, Cronbach’s α = 0.85) measures how selective children are with regard to trying new foods and enjoying a varied diet, and responses are scored on a 1–5 Likert scale ranging from “never” to “always.” Mean scores for the questions in each scale were generated. All 295 mothers completed the Perceived Child Weight and Concern about Child Weight questions from the CFQ. Four mothers failed to return for the second study visit, so only 291 mothers completed the CEBQ Food Fussiness questions.

### Statistical analysis

Descriptive statistics were calculated for the full sample as well as by maternal depressive symptoms, and bivariate analyses were completed using t-tests for continuous variables, Chi-square tests for categorical variables, and unadjusted Poisson regression for count data. We then ran multiple regression models for our adjusted analyses, predicting individual measures of maternal feeding practices from elevated maternal depressive symptoms (CES-D score ≥ 16). Linear regression was used for continuous outcomes, logistic regression for binary outcomes, and Poisson regression for count outcomes. Covariates, selected on the basis of previous research demonstrating associations with maternal feeding behaviors or attitudes, included: child sex [43] and food fussiness [44]; and maternal BMI [13], education [17], race/ethnicity [29,31], perceived child weight [13], and concern about child weight [13]. Additional variables were included as covariates based on theoretical considerations that they could influence the maternal-child feeding interaction: number of older siblings, maternal age, and single parent status.

We created separate models for each of the different measures of maternal feeding, with analysis limited to those with complete data for all covariates (sample sizes provided in parentheses): Perceived Responsibility (n = 289), Pressure to Eat (n = 289), Restriction (n = 288), Monitoring (n = 289), and Demandingness (n = 289) from questionnaires; authority, confidence, and investment (n = 287) from semi-structured narrative interviews; child always eating at kitchen/dining table, television always audible, and mother always eating with child for some portion of the meal (n = 267) from home meal observations; and total maternal encouragements and discouragements for familiar and unfamiliar foods (n = 226) from laboratory eating interactions. All models were re-run limiting the sample to those with child gestational age ≥ 37 weeks, which excluded 16 mother-child pairs with child gestational age between 35–37 weeks. Analyses were conducted using SAS 9.3 (SAS Institute, Cary, NC), and two-sided *P*-values of < 0.05 were considered statistically significant.

## Results

Mean child age was just under 6 years, and 52% of children were male (Table 4). Among mothers, 31% reported depressive symptoms above the threshold of CES-D score ≥ 16. Thirty-two percent of mothers were Hispanic and/or non-white, and among this group the breakdown was approximately 50% non-Hispanic black, 25% Hispanic (of any race), and 25% identifying as more than one race. Forty-eight percent of mothers reported no additional education beyond high school. The mean maternal BMI was 33.08, with standard deviation of 9.36.

**Table 4:** **Sociodemographic characteristics and feeding practices for full sample and by level of maternal depressive symptoms**^**1**^

		**Total**	**Lower level maternal depressive symptoms**^**2**^	**Elevated maternal depressive symptoms**^**3**^	***P*-value**^**4**^
		**n = 295**	**n = 204**	**n = 91**	
**Sociodemographic characteristics**					
Child age, months		70.83 ± 8.32	70.60 ± 8.09	71.33 ± 8.84	NS
Child sex male		152 (52)	104 (50.98)	48 (53)	NS
Number of older siblings		0.92 ± 1.01	0.90 ± 0.99	0.95 ± 1.06	NS
Maternal age, n = 294		30.99 ± 7.03	31.02 ± 6.87	30.93 ± 7.41	NS
Maternal BMI, n = 293		33.08 ± 9.36	32.60 ± 9.07	34.16 ± 9.95	NS
Maternal education ≤ high school		141 (48)	90 (44.12)	51 (56)	NS
Mother Hispanic/non-white		95 (32)	66 (32.35)	29 (32)	NS
Mother single parent		132 (45)	88 (43.14)	44 (48)	NS
**Feeding practices**					
*Questionnaires*					
Perceived responsibility		4.45 ± 0.65	4.46 ± 0.66	4.43 ± 0.65	NS
Pressure to eat		2.72 ± 1.07	2.61 ± 1.04	2.97 ± 1.11	<.01
Restriction, n = 294		3.31 ± 0.92	3.24 ± 0.94	3.48 ± 0.86	<.05
Monitoring		4.01 ± 1.03	3.99 ± 1.06	4.05 ± 0.99	NS
Demandingness		2.55 ± 0.55	2.49 ± 0.52	2.67 ± 0.60	<.01
Concern about child weight		1.72 ± 0.91	1.69 ± 0.95	1.80 ± 0.83	NS
Perceived child weight					NS
Underweight		54 (18)	34 (17)	20 (22)	
Normal		203 (69)	142 (70)	61 (67)	
Overweight		38 (13)	28 (14)	10 (11)	
Food Fussiness, n = 291		2.73 ± 0.77	2.70 ± 0.80	2.78 ± 0.67	NS
*Interviews, n = 293*					
Authority					< .001
Low		70 (24)	36 (18)	34 (38)	
High		223 (76)	167 (82)	56 (62)	
Confidence					NS
Not confident		65 (22)	40 (20)	25 (28)	
Confident		228 (78)	163 (80)	65 (72)	
Investment					NS
Removed		61 (21)	36 (18)	25 (28)	
Mildly		143 (49)	101 (50)	42 (47)	
Deeply		89 (30)	66 (33)	23 (26)	
*Home meal observations, n = 270*					
Child always eats at table		221 (82)	158 (85)	63 (75)	<.05
TV always audible during meal		76 (28)	44 (24)	32 (38)	<.05
Mother always eats with child		161 (60)	121 (65)	40 (48)	<.01
*Laboratory eating interactions, n = 228*					
Encouragements	Familiar food	4.46 ± 4.59	4.26 ± 4.30	4.84 ± 5.11	NS
	Unfamiliar food	6.96 ± 7.51	7.17 ± 7.34	6.56 ± 7.87	NS
Discouragements	Familiar food	1.96 ± 3.15	2.09 ± 3.39	1.69 ± 2.64	NS
	Unfamiliar food	1.24 ± 2.34	1.15 ± 2.22	1.40 ± 2.57	NS

^1^Total sample size n = 295 unless otherwise stated. Values expressed as “number of participants (%)” or “mean ± SD”. Percentages may not add up to 100% due to rounding. NS = Non-significant.

^2^Center for Epidemiologic Studies-Depression scale (CES-D) score < 16.

^3^CES-D score ≥ 16.

^4^P-values calculated using t-tests for continuous variables, Chi-square tests for categorical variables, and unadjusted Poisson regression for count data.

In bivariate analyses, there were no significant differences in sociodemographic characteristics when comparing mothers with elevated depressive symptoms to those with lower levels of depressive symptoms (Table 4). Mothers with elevated depressive symptoms self-reported more pressure to eat, restriction, and demandingness in feeding questionnaires. They were more likely to present as low authority in child feeding during the semi-structured narrative interview. In homes of mothers with elevated depressive symptoms, children were less likely to eat at the kitchen/dining table, the television was more likely audible during meals, and mothers were less likely to eat with their children. No behaviors from laboratory eating interactions differed significantly in bivariate analyses.

In our adjusted linear regression models of mothers’ questionnaire responses (Table 5), those with elevated depressive symptoms reported more pressure to eat (β = 0.29, p = 0.027) and more demandingness (β = 0.16, p = 0.017). There were no significant differences in maternal report of perceived responsibility, restriction, or monitoring between mothers with and without elevated depressive symptoms.

**Table 5:** **Adjusted linear regression models predicting maternal feeding practices from maternal depressive symptoms**^**1**^

**Outcome**	**Beta (95% CI)**
**Questionnaires**^**2**^	
Perceived responsibility	−0.06 (−0.22, 0.11)
Pressure to eat	0.29 (0.03, 0.54)
Restriction	0.22 (−0.01, 0.44)
Monitoring	0.04 (−0.22, 0.31)
Demandingness	0.16 (0.03, 0.29)

^1^Adjusted for: child sex, food fussiness, number of older siblings; and maternal age, BMI, education, race/ethnicity, single parent status, perceived child weight, and concern about child weight. All models are comparing mothers with elevated depressive symptoms (Center for Epidemiologic Studies-Depression scale (CES-D) score ≥16) to mothers with lower levels of depressive symptoms (CES-D score < 16).

^2^n = 289 with complete data for adjusted models for all questionnaire outcome measures, except Restriction (n = 288).

In our adjusted logistic regression models of mothers’ narrative interview data (Table 6), those with elevated depressive symptoms had approximately three times greater odds of presenting as low authority in child feeding (OR = 2.82, p = 0.001). There were no significant differences in maternal confidence or investment in comparing mothers with and without elevated depressive symptoms. In our adjusted logistic regression models of home meal observations (Table 6), the odds of the television being audible during mealtimes were higher (OR = 1.91, p = 0.034), while the odds of the mother eating with the child were lower (OR = 0.48, p = 0.012), in homes of mothers with elevated depressive symptoms. Elevated maternal depressive symptoms were not significantly associated with the odds of the child eating at the kitchen/dining table.

**Table 6:** **Adjusted logistic regression models predicting maternal feeding practices from maternal depressive symptoms**^**1**^

**Outcome**	**OR (95% CI)**
**Interviews**^**2**^	
Low authority	2.82 (1.55, 5.12)
Not confident	1.41 (0.76, 2.59)
Removed (vs. mild or deep investment)	1.68 (0.91, 3.13)
**Home meal observations**^**3**^	
Child always eats at table	0.60 (0.30, 1.21)
TV always audible during meal	1.91 (1.05, 3.48)
Mother always eats with child	0.48 (0.27, 0.85)

^1^Adjusted for: child sex, food fussiness, number of older siblings; and maternal age, BMI, education, race/ethnicity, single parent status, perceived child weight, and concern about child weight. All models are comparing mothers with elevated depressive symptoms (Center for Epidemiologic Studies-Depression scale (CES-D) score ≥16) to mothers with lower levels of depressive symptoms (CES-D score < 16).

^2^n = 287 with complete data for adjusted models for all interview outcome measures.

^3^n = 267 with complete data for adjusted models for all home meal observation outcome measures.

In our adjusted Poisson regression models of laboratory eating interactions (Table 7), there were no significant differences in rates of encouragement or discouragement for familiar or unfamiliar foods in comparing mothers with and without elevated depressive symptoms.

**Table 7:** **Adjusted Poisson regression models predicting maternal feeding practices from maternal depressive symptoms**^**1**^

**Outcome**		**Relative rate (95% CI)**
**Laboratory eating interactions**^**2**^		
Encouragements	Familiar foods	1.13 (0.85, 1.49)
	Unfamiliar foods	0.93 (0.70, 1.24)
Discouragements	Familiar foods	0.81 (0.52, 1.26)
	Unfamiliar foods	1.22 (0.73, 2.05)

^1^Adjusted for: child sex, food fussiness, number of older siblings; and maternal age, BMI, education, race/ethnicity, single parent status, perceived child weight, and concern about child weight. All models are comparing mothers with elevated depressive symptoms (Center for Epidemiologic Studies-Depression scale (CES-D) score ≥16) to mothers with lower levels of depressive symptoms (CES-D score < 16).

^2^n = 226 with complete data for adjusted models for all laboratory eating interaction outcome measures.

After all of the models were re-run limited to mother-child pairs with child gestational age ≥ 37 weeks, none of the results differed significantly.

## Discussion

This study of low-income mothers of 4- to 8-year-old children found that mothers reporting elevated depressive symptoms exhibited different child feeding practices than those with lower levels of depressive symptoms: they self-reported more pressure to eat and demandingness; were more likely to express low authority in child feeding during a semi-structured narrative interview; and at home, were more likely to have the television audible during meals and less likely to eat meals with their children. Findings were independent of potential confounders, including: child sex, food fussiness, number of older siblings; and maternal age, BMI, education, race/ethnicity, single parent status, perceived child weight, and concern about child weight.

Collectively, our findings suggest a less contingently responsive feeding style in mothers with elevated depressive symptoms. Contingent responsive feeding involves reciprocity between caregiver and child, where the caregiver provides appropriate guidance while recognizing child hunger and satiety cues [12]. In our study, some feeding practices associated with maternal depressive symptoms appeared controlling (more pressure to eat and demandingness), while others could be characterized as indulgent (lower authority narratives about feeding) or uninvolved (television audible during meals, mother not eating with child). Controlling, indulgent, and uninvolved feeding styles represent a lack of reciprocity between mother and child, and can be described as nonresponsive [45,46]. Nonresponsive feeding practices may increase risk for child overweight/obesity [12,47].

Our study expands on prior work by using multiple methods to assess maternal feeding practices, and we found the association between maternal depressive symptoms and feeding to be inconsistent across methodologies. Mothers with elevated depressive symptoms self-reported more pressure and demandingness, yet appeared lower authority in narrative interviews about child feeding and less engaged during home meal observations. Questionnaire, narrative interview, and video observation methods each have unique strengths and weaknesses. While our findings do not identify the ideal methodology for characterizing maternal feeding, they raise questions regarding the validity of relying on a single methodology for data collection.

Using self-report questionnaires, we found that mothers with elevated depressive symptoms reported more pressuring of their children to eat, while there was no association with restriction. These findings are consistent with other reports in the literature, which suggest that maternal depressive symptoms may be associated with pressuring children to eat [9], but are not associated with restrictive feeding practices [9,13-16]. Most studies have examined white and/or middle-class populations [9,10,13-15,22,23]. There have been only two self-report studies in populations comparable to our sample, and one was restricted to infants less than 13 months old [11]. The study most similar to ours reported differing results: It included 401 low-income mothers and their 5-year-old children, and found no association of maternal depressive symptoms with pressure to eat and an inverse association with restriction [3]. Given evidence that maternal feeding practices differ based on race/ethnicity [29,31], more research needs to be done in diverse populations to clarify the association of maternal depressive symptoms with pressuring and restrictive feeding practices.

Examining maternal feeding practices via alternative, non-questionnaire methods can help us better understand associations between maternal depressive symptoms and feeding. To our knowledge, there are no other published studies using interviews or videotaped home meal observations to examine these associations. We found that mothers with elevated depressive symptoms expressed lower authority in child feeding during a semi-structured narrative interview. During home mealtimes, elevated maternal depressive symptoms were positively associated with the television being audible and inversely associated with the mother eating with her child, suggesting a less engaged feeding style. These novel finding should be explored further.

Our study found that elevated maternal depressive symptoms were not associated with encouragement or discouragement of eating during interactions around food in the laboratory. Two prior studies observed standardized laboratory eating interactions to examine similar associations, and only one included low-income and minority mothers and children. This multi-site study of 1218 mother-child pairs in the United States found that maternal depressive symptoms were not associated with prompting feeding behaviors at child ages 15, 24, or 36 months [17]. In contrast, the other study examined a British sample of 58 mothers of 3- to 4-year-old children and found an association between maternal depressive symptoms and pressure to eat, use of incentives, and maternal vocalizations about food during the observed meal [10]. While evidence is limited, our findings support those of the first study, suggesting that self-report of controlling feeding practices may not be consistent with videotaped observations in mothers with depressive symptoms, as has previously been observed in non-depressed mothers [20,21].

We hypothesize several behavioral mechanisms to explain the feeding practices associated with elevated maternal depressive symptoms in our study. The association between depressive symptoms and self-report of controlling feeding practices, in contrast to interview and video observation findings, may be due to the preferential recall of negative events that has been well-described in depression [48]. Mothers with depressive symptoms may have enhanced recall of difficult feeding interactions eliciting more controlling feeding behaviors, as compared to mothers without depressive symptoms. The association between low parenting self-efficacy and depression [49,50] may contribute to descriptions of lower authority feeding styles among mothers with depressive symptoms. If mothers with depressive symptoms perceive themselves as less competent parents, they may be less likely to establish rules and expectations around child feeding because they do not anticipate that such measures will be effective. Findings of a less engaged feeding style among mothers with depressive symptoms during home meal observations may simply reflect the core symptoms of depression, including low energy and diminished pleasure in activities [51]. Depression can influence behavior in many ways, and it is important for clinicians to consider this when interacting with parents who may appear to be engaging in suboptimal feeding practices.

Our results should be interpreted in light of both our study’s strengths and limitations. The greatest strength of our study is that we characterized feeding in a detailed, multi-method manner, which allowed us to examine how associations between maternal depressive symptoms and maternal feeding practices vary depending on how feeding is measured. We also examined a more diverse, lower socioeconomic status population than most prior studies. Our study was limited in its cross-sectional design, which did not allow for assessment of temporality of associations. While we did control for many possible confounders in our analysis, it is possible that there were additional confounders for which we did not account. Finally, our results may not be generalizable to populations outside of low-income families in Southeastern Michigan.

## Conclusions

The findings from our study suggest that low-income mothers with elevated depressive symptoms exhibit less responsive feeding practices that may interfere with development of healthy child eating behaviors. Given that depression is common [52] and can be effectively treated among low-income women [53], it represents an important modifiable factor influencing both mother and child wellbeing. Prior studies have demonstrated that it is feasible for clinicians to screen mothers for depressive symptoms at well-child visits [54,55]. While further study is needed, our findings suggest that such screening may be an important component of counseling on healthy child feeding practices. Given inconsistencies in styles of feeding associated with maternal depressive symptoms across methodologies, researchers and clinicians should use caution in interpreting findings from studies relying on a single methodology to assess maternal feeding. Future investigations should consider multi-method assessment of maternal feeding practices and targeted comparisons of different methodologies.

## Abbreviations

BMI: Body mass index; CEBQ: Child eating behavior questionnaire; CES-D: Center for epidemiologic studies-depression scale; CFQ: Child feeding questionnaire; CFSQ: Caregiver’s feeding styles questionnaire; CI: Confidence interval; OR: Odds ratio; SD: Standard deviation.

## Competing interests

The authors declare that they have no competing interests.

## Authors’ contributions

KLR, ALM, KEP, and JCL designed the research; KLR, ALM, and JCL conducted the research; YC and NK analyzed the data; ANG, KLR, ALM, KEP, and JCL wrote the paper; JCL had primary responsibility for final content. All authors read and approved the final manuscript.

## Authors’ information

ANG is a resident physician in Obstetrics and Gynecology, University of North Carolina, Chapel Hill; at the time of the study, she was a student, University of Michigan Medical School, Ann Arbor. KLR is an Associate Research Scientist, Center for Human Growth and Development, University of Michigan; and Clinical Associate Professor, Department of Psychiatry, University of Michigan, Ann Arbor. ALM is an Assistant Research Professor, Department of Health Behavior and Health Education, University of Michigan School of Public Health; and Assistant Research Scientist, Center for Human Growth & Development, University of Michigan, Ann Arbor. KEP is a Professor, Human Nutrition Program, Department of Environmental Health Sciences, School of Public Health and Research Professor, Center for Human Growth and Development, University of Michigan, Ann Arbor and Adjunct Professor, Department of Nutrition, Harvard School of Public Health, Boston. YC is a PhD Candidate, Department of Biostatistics, University of Michigan School of Public Health, Ann Arbor. NK is a Research Scientist, Departments of Biostatistics and Bioinformatics, Center for Human Growth & Development, University of Michigan, Ann Arbor. JCL is an Associate Professor, Department of Pediatrics, University of Michigan Medical School; Associate Professor, Department of Environmental Health Sciences, University of Michigan School of Public Health; and Associate Research Professor, Center for Human Growth and Development, University of Michigan, Ann Arbor.
